# Prevalence and Psychological Impact of Acne Vulgaris in Female Undergraduate Medical Students of Rawalpindi and Islamabad, Pakistan

**DOI:** 10.7759/cureus.5722

**Published:** 2019-09-22

**Authors:** Osama Babar, Amen Mobeen

**Affiliations:** 1 Dermatology, Islamic International Medical College (Riphah International University), Rawalpindi, PAK

**Keywords:** acne vulgaris, psychological impact, dermatology life quality index (dlqi), female medical undergraduate students, prevalence, acne in pakistan, rawalpindi/islamabad

## Abstract

Background:

Acne vulgaris is a common skin disease, affecting approximately 9.4% of the world’s population, with considerable effect on the quality of life. According to a previously conducted study, the prevalence rate of acne in Pakistan was found to be 5%. And to this date, no reliable data is available about the prevalence of acne in Rawalpindi and Islamabad, Pakistan.

Objectives

To determine the prevalence of acne vulgaris and its psycho-social impact on female undergraduate medical students of Rawalpindi and Islamabad.

Methods

A cross-sectional study was conducted during the month of August 2019 among female undergraduate medical students from three randomly selected medical colleges of Rawalpindi and Islamabad. The diagnosed cases of acne vulgaris were assessed by using the Dermatology Life Quality Index (DLQI). The collected data were then analyzed using SPSS version 20 (IBM Corp., Armonk, NY, US).

Results

The prevalence of acne vulgaris was found to be 14.47% in female undergraduate medical students of Rawalpindi and Islamabad. Sixty percent (n=99) were found to have itchy sores and stinging skin, 66.7% (n=110) were embarrassed by their acne-prone skin, and the social activity of 60% (n=99) of the participants was affected by their active acne. Of the students, 73.9% were not affected by their acne while studying or working. Around 61.2% (n=101) complained that their acne treatment was a problem and hiding it took time or made a mess.

Two percent showed a severe impact, with 14% having very large, 44% moderate, 30% low, and the remaining 10% with no effect of acne in their psychosocial functioning.

Conclusion

Acne vulgaris is a chronic skin disease that considerably affects the psychosocial functioning of female undergraduate medical students. A holistic approach in treating acne requires the participation of a dermatologist and mental health professional.

## Introduction

Acne vulgaris is a chronic dermatological disease characterized by inflammatory changes in the pilosebaceous glands of the skin resulting in the formation of comedones, pustules, papules, nodules, and cysts. It is commonly associated with the bacterium Cutibacterium acnes (formerly Propionibacterium acnes ) [1].

The Global Burden of Disease project has estimated the prevalence of acne to be 9.4%, ranking it as the eighth most prevalent disease worldwide. The project found a prevalence of around 5% in Pakistan [2]. The frequency of acne vulgaris has been reported in many studies but there is a lack of data regarding the prevalence of the disease and its psychosocial impact in adolescents and young adults. On comparing both genders, it was found to have a greater psychosocial impact on females [3-5].

Acne vulgaris is the most common type of acne and carries a significant burden of disease. It may cause severe discomfort, permanent facial scarring, emotional and physical distress, occupational consequences, and potential psychiatric disturbances like depressive disorders and suicidal tendencies [2]. It has also been found to have a negative influence on people who intend to participate in sports [6].

The measurement of the quality of life of acne sufferers has been acknowledged as being of pivotal value in the assessment and management of the disease [7]. Previously conducted studies have found immense improvement in the symptoms and aforementioned disturbances after the treatment of the disease [8].

Acne and its impact have been well-researched and studied in various parts of the world, including the USA, Europe, and Korea. However, data from South East Asian countries like Pakistan remains scanty [4]. Moreover, there is very limited data on adolescents and adults [1].

## Materials and methods

Research design

A cross-sectional study was conducted during the month of August 2019, among female undergraduate medical students from three randomly selected medical institutes of Rawalpindi and Islamabad.

Sample size and data collection

Only female medical students were considered for this respective study. Out of a total of 1140 female students, 165 were diagnosed cases of acne. The medical students were assumed to be aware of acne because of the extensive teaching of dermatology in their curriculum. The diagnosed cases were further assessed by the Dermatology Life Quality Index (DLQI). The absent students were excluded from this study.

Before the data collection, it was announced that the students will be surveyed and were explained the vitality of the research. The confidentiality of the participants was also ensured.

The diagnosed participants were then asked to fill out the Dermatology Life Quality Index (DLQI) to evaluate the psychosocial impact that acne had on their lives over the past two weeks. The English version of the DLQI was chosen. The questionnaire comprises 10 questions about acne symptoms, related feelings and influence in daily and social activities, type of clothing, playing sports, job and education, in interpersonal and sexual relationships and treatment. Every question has four options and each option on the DLQI is given a particular score. “Very much” (score three), "a lot" (score two), "a little" (score one), and both "not at all" and "not relevant" are scored zero. Each individually filled DLQI score was totaled and a final score was given. A high total score was an indicator of the poor quality of life of the participant due to the disease. The effect of the disease was divided into different groups according to the total score: (0-1) without any effect, (2-5) low effect, (6-10) moderate effect, (11-20) high effect, and (21-30) severe effect [9].

Ethical considerations

Ethical approval for this study was obtained from the Ethics Review Committee of Islamic International Medical College trust. All the participants from the three medical institutes were oriented on the topic of the study. Verbal consent was taken from everyone who filled out the DLQI. Furthermore, it was also mentioned in the DLQI that the personal information of all the participants would be kept highly confidential.

Statistical analysis

Data for this study were analyzed by using the SPSS Statistics version 20 (IBM Corp., Armonk, NY, USA).

## Results

A sample size of 216 students was calculated using the EpiData Software (Centers for Disease Control and Prevention, Georgia, US), which included diagnosed cases of acne along with acne-free students.

Our sample size was 1140 students from which 216 were diagnosed cases of acne vulgaris and 924 were disease-free. A prevalence rate of 14.47% (n=216) was calculated from this data. Out of the 216 questionnaires filled out, only 165 were analyzed to determine the psychosocial impact of the disease, giving a response rate of 76.389%. This was due to the incomplete submission of questionnaires.

A total of 60% (n=99) participants complained of itchy sores and painful stinging skin with 3% (n=5) very much, 16.4% (n=27) a lot, 40.6% (n=67) a little, and 40% (n=66) not at all. Sixty-six point seven percent (n=110) were embarrassed and self-conscious because of their acne-prone skin, with 7.9% (n=13) very much, 32.7% (n=54) a lot, 26.1% (n=43) a little, and 33.3% (n=55) not at all. More than half (53.3%, n= 55) of the participants were not worried about acne while shopping. Approximately half (50.3%, n=83) of the participants reported that their clothes were not influenced by their skin. The social and leisure activities of 60% (n=99) participants were affected by their acne with 9.7% (n=16) very much, 14.5% (n=24) a lot, 35.8% (n=59) a little, and 40% (n=66) not at all. A majority of the participants 67.3% (n=111) were not affected by their acne in playing sports. Moreover, 73.9% (n=122) participants were not affected by acne while studying and working and more than half (58.8%, n=97) of them did not have their acne influence their relationships with their partners and close friends. Eighty-four point eight (n=140) did not have any sexual difficulties due to their acne while 7.9% (n=13) had little, 6% (n=10) a lot, and 1.2% (n=2) had very many difficulties. Around 61.2% (n=101) complained that their acne treatment and masking was a problem and took time or made a mess, with 38.2% (n=63) a little, 10.3% (n=17) a lot, 12.7% (n=21) very much, and 38.8% (n=64) not at all. (Table 1).

**Table 1:** Descriptive analysis of the Dermatology Life Quality Index (DLQI) in female undergraduate medical students of Rawalpindi and Islamabad having acne.

Table 1: Descriptive Analysis of the Dermatology Life Quality Index (DLQI) of Female Students with Acne			
		n	%
Itchy, sore and painful/stinging skin	Not at all/ Not relevant	66	40%
	A little	67	40.6%
	A lot	27	16.4%
	Very Much	5	3%
Embarrassed or self-conscious	Not at all/ Not relevant	55	33.3%
	A little	43	26.1%
	A lot	54	32.7%
	Very Much	13	7.9%
Skin interfered during shopping, looking after home or garden	Not at all/ Not relevant	88	53.3%
	A little	48	29.1%
	A lot	26	15.8%
	Very Much	3	1.8%
Skin influenced the clothes	Not at all/ Not relevant	83	50.3%
	A little	55	33.3%
	A lot	23	13.9%
	Very Much	4	2.4%
Skin affected social or leisure activities	Not at all/ Not relevant	66	40%
	A little	59	35.8%
	A lot	24	14.5%
	Very Much	16	9.7%
Skin made it difficult to play sports	Not at all/ Not relevant	111	67.3%
	A little	30	18.2%
	A lot	12	7.3%
	Very Much	12	7.3%
Skin prevented from working or studying	Not at all/ Not relevant	122	73.9%
	A little	26	15.8%
	A lot	3	1.8%
	Very Much	14	8.5%
Skin created problems with partner or friends	Not at all/ Not relevant	97	58.8%
	A little	44	26.7%
	A lot	20	12.1%
	Very Much	4	2.4%
Skin caused sexual difficulties	Not at all/ Not relevant	140	84.8%
	A little	13	7.9%
	A lot	10	6%
	Very Much	2	1.2%
Skin treatment was a problem e.g taking time or making a mess	Not at all/ Not relevant	64	38.8%
	A little	63	38.2%
	A lot	17	10.3%
	Very Much	21	12.7%

**Figure 1:**
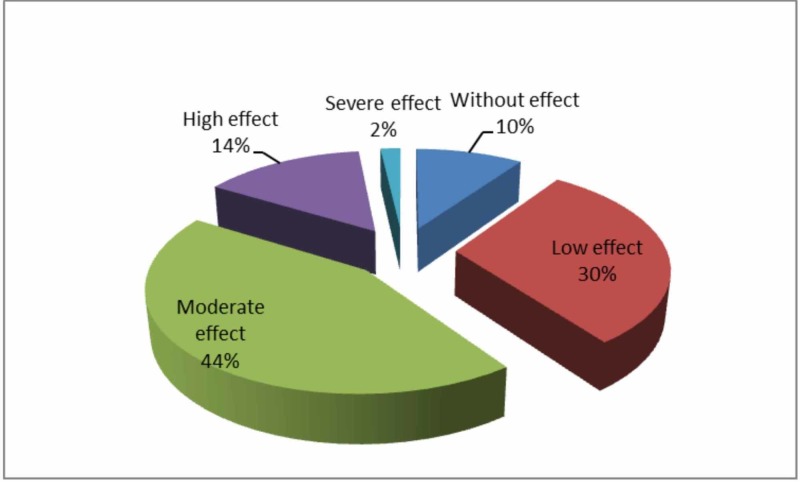
Effect of acne on the psycho-social functioning of female undergraduate medical students of Rawalpindi and Islamabad.

The psychosocial impact of acne on the participants was calculated by using the final scores of the DLQI. Two percent showed a severe impact, with 14% having very large, 44% moderate, 30% low, and the remaining 10% with no effect of acne on their psychosocial functioning. (Figure 1).

## Discussion

Acne is a chronic inflammatory disease, and its prevalence varies in adolescents and adults among different countries and ethnic groups. Around 9.4% of the world's population suffers from acne, making it the eighth-most prevalent disease worldwide [2].

In the US, acne is seen in 85% of teenagers [10]. An overall prevalence of 60.7% was seen among adolescents of Turkey [11]. In Malaysia, a study conducted among medical students reported a prevalence of 68.1% [12]. Whereas another community-based study carried out in China reported an acne prevalence of 38% among females aged 15-19 years [13]. A research carried out in Saudi Arabia found an overall prevalence of 14.3% among female secondary school students [14]. Most of these studies have been conducted in secondary school or high school students, with very few conducted in college and university students [4,12,15]. Our study focused on finding the prevalence of acne in female medical college students.

Sixty percent of the cases from this study reported having physical symptoms due to acne in the form of itchy sores or burning and stinging skin. According to Tasoula et al., 22.8% of the cases who had facial acne and 31.5% of those with back acne suffered from itchiness [16]. In another study by Reich et al., about 50% of the cases reported itchiness as a physical symptom [17]. However, the exact nature of each physical manifestation was beyond the scope of this study.

Sixty-six point seven percent of the cases reported feeling embarrassed or self-conscious because of their acne. These results were consistent with another study [16]. A study by Ogedegbe found that 43.7% of the patients actually felt frustrated and embarrassed because of their acne papules and nodules [7]. In the Magin et al. study about the psychological sequelae of acne vulgaris, it was found that acne vulgaris has a negative influence on the overall self-esteem and image of the sufferers [18].

Forty-six point seven percent of the females reported that their acne interfered in their daily activities like shopping and looking after their homes. In a 2016 study by Hazarika and Archana, about 69% of the patients agreed to having faced problems in their routine activities because of their acne. A link was established between frustration, anger, and acne. It is, therefore, deducible that problems in their daily activities due to acne maybe because of evasive behavior, annoyance, and anger [6].

It has been well-established that one's appearance is often acknowledged through their clothing, making it an integral part of social acceptance [19]. Forty-nine point seven percent of the patients reported that acne influenced their choice of clothing. In the 2014 study by Ogedegbe and Henshaw, 14.4% of adolescents avoided wearing clothes that revealed their extra facial acne [5].

It was found that the social life and leisure activities of 60% of the females were affected by acne, and they avoided going out to public places and interacting in community-based events during acne flare-ups. Yolaç et al. have also reported that social withdrawal and anxiety are major sequelae of acne among female patients [20]. Another study by Magin et al. reported avoidant behavior in many of the subjects, ultimately leading to the development of avoidant personality traits [18].

In a study, Tasoula et al. found that 14.4% of the patients reported having problems in their sports activities because of their acne [16]. We found that 32.7% of the female participants found difficulties of various degrees in sports and related activities due to their acne.

The work and studies of 26.1% of the females in this study were affected by their acne. These results were consistent with another recent study conducted in 2018 where most of the subjects reported that acne did not have a significant influence on their work or studies. However, some subjects felt that their attention on work was diverted during acute acne flare-ups, and a couple of students even had to take a day off from school because of their acne while adults reportedly had to make excuses from work during times when their acne would be at its worse [21].

We found that the relationships of 41.2% of the female students were affected by their acne. According to Magin et al., the media’s portrayal of people with flawless skin has led to a false perception among its audience, leading them to believe that acne is unsanitary and contagious and clear skin is essential. This has contributed to acne patients feeling a lack of desirability [18].

Many females believe that their physical appearance has an immense influence on getting jobs and sexual partners [5]. We found that 15.2% of our patients had difficulties with their sexual partners while 2% found severe difficulty. Most participants marked the question as not relevant, possibly because they were unmarried and sexual relations outside marriage in Pakistan are a religious and criminal offense. A further, detailed analysis was beyond the scope of this study. It was found similarly low in another research having to do with the fact that most women found it embarrassing to answer questions regarding their sexual activities [22].

People suffering from acne usually buy over-the-counter acne medications before visiting a certified dermatologist [16]. Makeup application and camouflaging techniques to hide acne have led to gratification and improved quality of life for many females [23]. Subjects, however, had to spend a long time every day in order to hide their lesions and scar marks. Complaints of acne being time-consuming and skin treatment being messy were consistent with 61.2% of the females.

In 90% of the participants in this study, acne affected their psychosocial functioning to varying degrees, with 2% severely affected and 14% highly affected. A large percentage, however, remained moderately affected, amounting to 44%, while 30% had low and 10% no effect on their psychosocial functioning.

Limitations

The study was conducted with female undergraduate medical students and further research needs to be done on male students and the overall prevalence as well. The data collected relied on self-reported information and depended on its subjects for authenticity. The study based its results on previously diagnosed cases of acne, assuming their knowledge and awareness of the disease due to dermatology-dedicated modules in their course of study, which may have led to a slight underestimation of the prevalence due to undetected cases, however unlikely.

A detailed analysis of the psychosocial management and its effects on improving dysfunction due to acne was beyond the scope of this research and needs further investigation.

## Conclusions

The prevalence rate of acne vulgaris is 14.47% in female undergraduate medical students of Rawalpindi and Islamabad, Pakistan. It has a significant impact on their psychosocial functioning, amounting to 90%, with a variable degree of dysfunction. It causes feelings of embarrassment, difficulties in studies and work, disruption in daily social activities, and impairment of interpersonal relationships, along with its physical symptoms. Since acne is not just a disease of the skin and causes significant psychosocial impairment, treating skin lesions alone isn't sufficient. DLQI is a good tool and should be used by dermatologists and general practitioners in evaluating the patients. Psychiatrists, psychologists, and other mental health professionals should be included in the treatment plan for acne in treating psychosocial dysfunction if warranted by the DLQI.

Doctors need to be educated on the psychosocial implications of acne, and schools and colleges should have support groups and ease of access to mental health professionals for students with diseases like acne that are likely to lead to psychosocial distress and impaired functioning.
